# Barriers to effective prescribing in older adults: applying the theoretical domains framework in the ambulatory setting – a scoping review

**DOI:** 10.1186/s12877-020-01766-7

**Published:** 2020-11-09

**Authors:** Sabrina Lau, Penny Lun, Wendy Ang, Keng Teng Tan, Yew Yoong Ding

**Affiliations:** 1 Department of Geriatric Medicine, Tan Tock Seng Hospital, TTSH Annex 2, Level 3, 11 Jalan Tan Tock Seng, Singapore, 308433 Singapore; 2 Geriatric Education & Research Institute, Singapore, Singapore; 3 Pharmacy, Changi General Hospital, Singapore, Singapore; 4 Pharmacy, Tan Tock Seng Hospital, Singapore, Singapore

**Keywords:** Barriers to effective prescribing in older adults, Multimorbidity, Ambulatory, Outpatient

## Abstract

**Background:**

As the population ages, potentially inappropriate prescribing (PIP) in the older adults may become increasingly prevalent. This undermines patient safety and creates a potential source of major morbidity and mortality. Understanding the factors that influence prescribing behaviour may allow development of interventions to reduce PIP. The aim of this study is to apply the Theoretical Domains Framework (TDF) to explore barriers to effective prescribing for older adults in the ambulatory setting.

**Methods:**

A scoping review was performed based on the five-stage methodological framework developed by Arksey and O’Malley. From 30 Aug 2018 to 5 Sep 2018, we conducted our search on PubMed, CINAHL, EMBASE, the Cochrane Database of Systematic Reviews, and Web of Science. We also searched five electronic journals, Google and Google Scholar to identify additional sources and grey literature. Two reviewers applied eligibility criteria to the title and abstract screening, followed by full text screening, before systematically charting the data.

**Results:**

A total of 5731 articles were screened. Twenty-nine studies met the selection criteria for qualitative analysis. We mapped our results using the 14-domain TDF, eventually identifying 10 domains of interest for barriers to effective prescribing. Of these, significant domains include physician-related factors such as “Knowledge”, “Skills”, and “Social/Professional Role and Identity”; issues with “Environmental Context and Resources”; and the impact of “Social Influences” and “Emotion” on prescribing behaviour.

**Conclusion:**

The TDF elicited multiple domains which both independently and collectively lead to barriers to effective prescribing for older adults in the ambulatory setting. Changing the prescribing climate will thus require interventions targeting multiple stakeholders, including physicians, patients and hospital/clinic systems. Further work is needed to explore individual domains and guide development of frameworks to aid guide prescribing for older adults in the ambulatory setting.

**Supplementary Information:**

The online version contains supplementary material available at 10.1186/s12877-020-01766-7.

## Background

The aging population brings with it an increasing number of older adults (aged 65 years and above) living with chronic disease and taking medications on a regular basis. Compared to younger individuals, older adults are at increased risk for developing drug-related complications due to a multitude of reasons including frailty, multi-morbidity, altered drug pharmacokinetics and pharmacodynamics, as well as a higher proportion of polypharmacy [1]. This predisposes the older adult to an increased risk of potentially inappropriate prescribing (PIP).

PIP describes the use of medications where the actual or potential harms of therapy outweigh the benefits, and encompasses both potentially inappropriate medications (PIMs) and potential prescribing omission (PPOs) [2]. PIP increases the risk of undesirable clinical consequences including adverse drug events (ADEs), functional decline, falls, cognitive impairment, medication non-adherence, and mortality [3]. Multiple screening tools have been developed to identify PIMs and PPOs in older adults, including The Improving Prescribing in the Elderly Tool, The Medication Appropriate Index, Beers’ criteria, and Screening Tool of Older Person’s Prescriptions (STOPP) and Screening Tool to Alert doctors to Right Treatment (START) [4].

Despite these tools, PIP remains a significant problem worldwide, with studies estimating the prevalence of PIP in older adults between 31 and 73% [5–8]. Although certain factors (e.g. clinical complexity, conflict between patient and physician’s preferences) may be applicable across all settings, we hypothesize that there exists unique barriers to effective prescribing depending on the type of practice (e.g. inpatient vs. outpatient, primary care clinics vs. specialist clinics, rural vs. urban). For this study, we chose to focus on the outpatient or ambulatory care setting, where physicians may experience more time constraints during each individual patient encounter, lack of support from institution-based prescribing algorithms or pharmacist-led medication reviews, and the need to juggle medications from multiple prescribers [9–11]. Obtaining an in-depth understanding of the factors that influence physicians’ prescribing behaviour may allow development of interventions to reduce PIP.

The prescribing framework in Singapore has long-centred on the physician as the key source of prescribing and medication review in both the inpatient and ambulatory care settings. In 2018, Singapore launched the National Collaborative Prescribing Programme [12], a three-month programme that prepares pharmacists and advance practice nurses to obtain certification as collaborative prescribing practitioners who may prescribe medications under a Collaborative Practice Agreement with a medical practitioner. At present, these capabilities are subspecialty-specific (e.g. heart failure, renal failure) and would not be applicable to the overarching theme of this scoping review for prescribing in older adults.

This study thus aims to explore barriers to effective physician prescribing for older adults in the ambulatory setting. This review also serves as part of a proof-of-concept study in Phase 1 of an extended 3-phase project to improve prescribing for older adults at outpatient clinics in public hospitals in Singapore.

## Methods

To capture barriers reported by physicians without placing a limit on the scope or nature of studies, a scoping review was selected over a systematic review. In line with the goals of scoping reviews, quality of evidence and risk of bias were not assessed [13]. We adopted the five-stage methodological framework developed by Arksey and O’Malley [13], with advancements proposed by Levac, Colquhoun and O’Brien [14] and the Joanna Briggs Institute (JBI) [15].

### Stage 1: identifying the research question

Our aim is to map barriers experienced by physicians when they are prescribing for older adults with multi-morbidity. As the results will eventually help to inform formulation of an outpatient collaborative care intervention, we focused our search on studies conducted in the ambulatory setting including both primary care and specialty ambulatory care (i.e. hospital outpatient clinics, specialist clinics, and primary care clinics). Hence, our research question was finalized as:

What are the key barriers to appropriate prescribing for older adults receiving ambulatory care?

### Stage 2: identifying relevant studies

JBI’s three-step search strategy was adapted [15], with an initial limited search conducted in PubMed by one of the reviewers (SL). A list of relevant articles was identified and an analysis on the index terms and MeSH terms was performed to identify relevant search terms. In addition, JBI’s mnemonic PCC (population, concept, and context) [15] was utilized to finalize our search strategy, with guidance from a librarian. Table 1 shows a summary of the search terms.

**Table 1:** Summary of search terms

	Keywords (MeSH terms and text word)
**Population**	Aged, older adult(s), older patient(s), older person(s), older people, elderly, seniors
**Concept**	Inappropriate prescribing, drug prescriptions, practice patterns (physicians), clinical practice pattern(s), prescribing, deprescribing, deprescription, polypharmacy AND barrier(s), challenge(s) and difficulty/difficulties
**Context**	Ambulatory care, primary health care, outpatient, clinic(s), primary care

In the second step of the search, our full search strategy was applied across the following databases from 30 Aug 2018 to 5 Sep 2018: PubMed, The Cochrane Database of Systematic Reviews (CDSR), Embase, Web of Science and Cumulative Index to Nursing and Allied Health Literature (CINAHL). The full search strategy for the peer-reviewed databases is provided in Additional file 1.

Grey literature searches were conducted using Google and Google Scholar to capture non peer-reviewed publications on the subject. We reviewed the first 50 titles/websites that were displayed, sorted by relevance and limiting the publication date from 1998 onwards. In addition, we also searched electronic databases of the following five journals relevant to our topic, using limited key words: Age and Aging, Archives of Gerontology and Geriatrics, BMC Geriatrics, Gerontology Series A and Journal of the American Geriatrics Society. In addition, reference lists of the included studies were also searched. This last step was recommended in JBI’s three-step search strategy [15].

### Stage 3: study selection

Two reviewers (SL and DYY) who are practicing clinicians independently completed the first stage title and abstract screening, resulting in a total of 45 eligible studies for the second-stage full text screening. Twenty-nine studies were found to be eligible for inclusion, following full text screening by the same reviewers. Conflicts were resolved through discussion. The two-stage screening process was managed in Covidence [16], an online systematic review software. Table 2 shows the eligibility criteria used for screening.

**Table 2:** Eligibility criteria for scoping review

	Inclusion	Exclusion
Population	patients 65 years and older	children, adolescents and adults younger than 65 years
Concept	prescribing by physicians, barriers associated with general prescribing	prescribing by pharmacists or nurse practitioners, prescribing restricted to specific diseases or specific medication
Context	outpatient care including primary care	inpatient care, long term care
Others	–	study protocols

Studies which involved patients aged less than 65 years or only non-physician prescribers were automatically excluded from this scoping review. We included one study by Carthy et al. [17] which did not specify any patient age group as it explored an in-depth discussion of our topic of interest with the intended concept and context. We also included studies which featured both physician and non-physician prescribers, so as to enrich the thematic analysis and not prematurely exclude this source of data which incorporates our focus (i.e. physician prescribing).

### Stage 4: charting the data

One of the reviewers (SL) performed data extraction, charting the following information: Authors, year, country of origin, aims and purposes of the study, study population, sample size, methods of the studies and key findings on barriers identified by physicians. The second reviewer (DYY) validated the extracted data and made suggestions for changes and additions, with agreement from the first reviewer (SL).

### Stage 5: collating, summarising and reporting the results

Barriers identified in the studies were mapped to the Theoretical Domains Framework (TDF) proposed by Michie and colleagues [18]. The TDF synthesizes constructs drawn from 33 psychological theories relating to behaviour and behaviour change, and summarises them into 14 domains that were validated in 2012 [19]. The domains broadly capture influences of cognition, emotions, social and environmental factors that impact one’s behaviour [20].

The barriers were extracted and first mapped to the 14 domains in the TDF by the first reviewer (SL). The second reviewer (DYY) cross-checked and made suggestions, which was then discussed and agreed on with the first reviewer (SL). The results were subsequently shared and discussed with the rest of the authors, and finalised after several rounds of iterations. The flow of the process is reported using the PRISMA flow diagram [21].

## Results

**Fig. 1:**
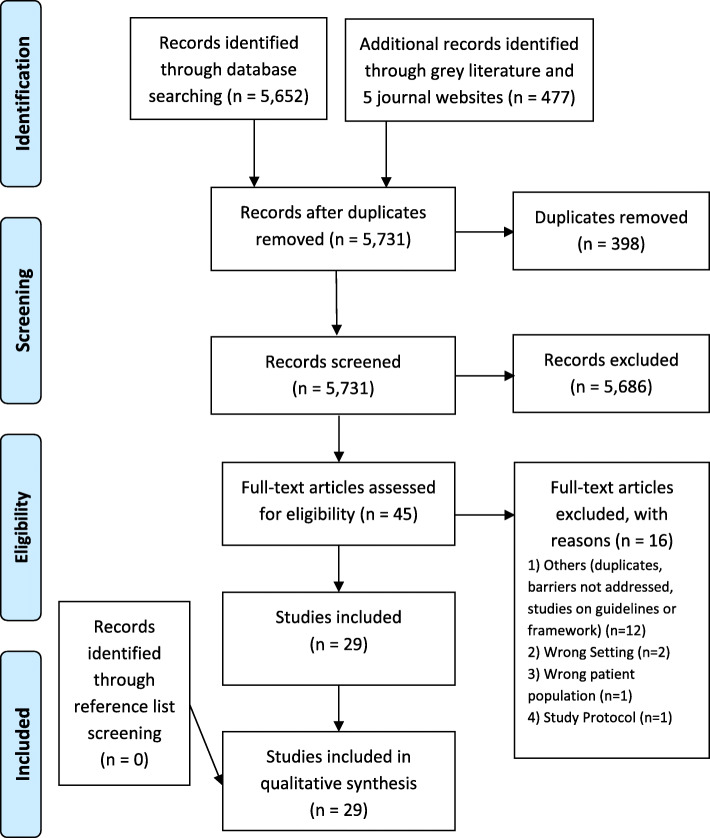
PRISMA 2009 Flow Diagram [21]

Our search yielded 5731 abstracts, of which 45 full-text articles were assessed for eligibility, and an eventual 29 articles were included in the qualitative synthesis (Fig. 1).

Barriers to effective prescribing in older adults were mapped to the TDF and categorised into major themes and constructs. The barriers identified mapped to the following 10 domains: knowledge; skills; social/professional roles and identity; beliefs about capability; beliefs about consequences; intentions; memory, attention and decision process; environmental contexts and resources; social influences; emotions. There were 4 TDF domains that the identified barriers did not map to: optimism; reinforcement; goals; behaviour regulation. This observation is not unexpected, as the nature of our research question (i.e. barriers to prescribing) is less likely to be associated with the more positive domains such as optimism and goals.

The identified domains were further subdivided based on their respective stakeholders (e.g. patient, physician, healthcare system) where appropriate so as to more effectively target interventions. Table 3 shows a summary of the studies selected, while Table 4 shows the results of our scoping review based on the TDF. It is here that we begin to appreciate the unique challenges of prescribing in older adults with multimorbidity, including medical complexity, patients’ own expectations and beliefs, and challenges with evidence-based guidelines often developed for a younger patient population with less multimorbidity. In the ambulatory setting, challenges faced by physicians include time and resource constraints, concerns on coordination of care and inter-professional relationships (especially in the context of multiple providers for a single patient), as well as anxiety and fear in a multitude of unknowns.

**Table 3:** Studies included in qualitative synthesis (*n* = 29) [10, 11, 17, 22–47]

No.	Authors	Year	Country of origin	Study population	Study methods
1	AlRasheed MM, Alhawassi TM, Alanazi A et al.	2018	Saudi Arabia	Family medicine physicians (n = 15)	Focus group discussions
2	Anderson K, Stowasser D, Freeman C, Scott I	2014	–	Systematic review of studies (*n* = 21)	Qualitative systematic review (PubMed, EMBASE, Scopus, PsycINFO, CINAHL and INFORMIT)
3	Anderson K, Foster M, Freeman C et al.	2017	Australia	General practitioners (*n* = 32), consultant pharmacists (n = 15)	Focus group discussions
4	Anthierens S, Tansens A, Petrovic M, Christiaens T	2010	Belgium	General practitioners (*n* = 65)	Semi-structured interviews
5	Bokhof B, Junius-Walker U	2016	–	Systematic review of studies (*n* = 14)	Qualitative systematic review (PubMed, Cochrane Library, Web of Science Core Collection and Scopus)
6	Cadogan CA, Ryan C, Francis JJ et al.	2015	Northern Ireland	General practitioners (n = 15), pharmacists (*n* = 15)	Semi-structured interviews
7	Cadogan CA, Ryan C, Gormley GJ et al.	2015	Northern Ireland	General practitioners (n = 14)	Semi-structured interviews
8	Carthy P, Harvey I, Brawn R, Watkins C	2000	United Kingdom	General practitioners (*n* = 17)	Semi-structured interviews
9	Clyne B, Cooper JA, Hughes CM et al.	2016	Ireland	General practitioners (n = 17)	Semi-structured interviews
10	Cullinan S, O’Mahony D, Fleming A, Byrne S.	2014	–	Systematic review of studies (*n* = 7)	Qualitative systematic review (PubMed, Embase, CINAHL and Web of Knowledge)
11	Cullinan S, Hansen CR, Byrne S et al.	2017	–	–	Review article
12	Djatche L, Lee S, Singer D et al.	2018	Italy	Primary care physicians (*n* = 160)	Questionnaire survey
13	Fried TR, Tinetti ME, Iannone L	2011	United States of America (USA)	Primary care clinicians (*n* = 40)	Focus group discussions
14	Lee PR, Boyd C, Green A	2018	USA	Primary care physicians (*n* = 12), specialist clinicians (*n* = 8)	Semi-structured interviews
15	Maio V, Jutkowitz E, Herrera K et al.	2011	Italy	Primary care physicians (*n* = 155)	Questionnaire survey
16	Mc Namara KP, Breken BD, Alzubaidi HT et al.	2017	Australia	Healthcare professionals (*n* = 26) ^a^medical, dentistry, nursing, pharmacy, allied health	Semi-structured interviews
17	Milos V, Westerlund T, Midlov P, Strandberg EL	2014	Sweden	General practitioners (n = 17)	Focus group discussions
18	Moen J, Norrgard S, Antonov K et al.	2010	Sweden	General practitioners (*n* = 31)	Focus group discussions
19	Newby C, Venditto A	2014	–	–	Clinical vignette session
20	Pohontsch NJ, Heser K, Loeffler A et al.	2017	Germany	General practitioners (*n* = 47)	Semi-structured interviews
21	Raae-Hansen C, Byrne S, O’Mahony D et al.	2017	–	Systematic review of studies (*n* = 10)	Qualitative systematic review (PubMed, CINAHL and Academic Search Complete)
22	Ramaswamy R, Maio V, Diamond JJ et al.	2011	USA	Residents and attending doctors (*n* = 89) ^a^Family Medicine, Internal Medicine, Geriatrics, Sports Medicine	Questionnaire survey
23	Riordan DO, Byrne S, Fleming A et al.	2017	Ireland	General practitioners (n = 16)	Semi-structured interviews
24	Roumie CL, Elasy TA, Wallston KA et al.	2007	USA	Primary care providers (*n* = 23)	Questionnaire survey
25	Schuling J, Gebben H, Veehof LJG, Haaijer-Ruskamp FM	2012	The Netherlands	General practitioners (n = 12)	Focus group discussions
26	Sellappans R, Lai PS, Ng CJ	2015	Malaysia	Family Medicine trainees (n = 14), service medical officers (*n* = 5)	Focus group discussions
27	Sinnige J, Korevaar JC, van Lieshout J et al.	2016	The Netherlands	General practitioners (n = 12)	Focus group discussions
28	Sinnott C, Mc Hugh S, Boyce MB, Bradley CP	2015	Ireland	General practitioners (*n* = 20)	Semi-structured interviews
29	Wallis KA, Andrews A, Henderson M	2017	New Zealand	Primary care physicians (*n* = 24)	Semi-structured interviews

**Table 4:** Scoping Review – Barriers to Effective Prescribing in Older Adults

Domain	Constructs	Barriers to Effective Prescribing
**Knowledge**	• Scientific knowledge • Procedural knowledge • Knowledge of task environment	**[Physician] Medical complexity** • Multimorbidity, potential interactions between diseases and medications • Polypharmacy, which increases difficulty in rationalizing and deprescribing medications • Increased risk of ADEs or drug-drug interactions • Difficulty in distinguishing between new complaints and medication side effects • Clinical uncertainty • Uncertainty in weighing unmeasurable harms and benefits **[Physician] Lack of knowledge or awareness** • Lack of awareness of PIP or PIMs • Poor insight into the term and the process of deprescribing • Lack of awareness of prescribing cost differences between care settings • Physicians’ shortcomings in their pharmacological knowledge • Doubts associated with potential ADEs and treatment of older adults • Lack of formal education on prescribing for older adults • Lack of up-to-date knowledge **[Patient] Lack of knowledge / poor healthcare literacy** • Patients do not understand what medications they are taking • Patients do not inform GPs about their medication intake or side effects • Patients may be more likely to report symptoms to hospital specialists rather than GPs • Unintentional withholding of ADEs because they attribute these to ageing rather than side effects of medications
**Skills**	• Skills • Skills development • Competence • Ability • Interpersonal skills • Practice • Skill assessment	**[Physician] Lack of skills and confidence** • Physician not comfortable with deprescribing (e.g. particularly when not the original prescriber) • Lack of confidence and clinical experience in managing older adult patients • Lack of research, education and training to care for this specific group of patients **[Physician] Challenges to discussion with patients** • Physicians are reluctant to talk to patients about their life expectancy • Problems with incorporating patients’ prognoses into decisions about therapy appropriateness • Difficulty in communicating risk to patients **[Patient] Non-adherence to medications or visits** • Lack of adherence to medications, or self-titration of medications • Usage of over-the-counter and traditional medications (e.g. often without informing the primary physician) • Non-adherence to clinic visits • Choosing to ‘doctor hop’ or ‘pharmacy hop’
**Social/Professional Role and Identity**	• Professional identify • Professional role • Social identity • Identity / group identity • Professional boundaries • Professional confidence • Leadership • Organizational commitment	**[Physician] Paternalistic doctor-patient relationship** • Physicians imposing their own beliefs onto the patient without consideration for the latter **[Physician] [System] Role dilemma** • Dilemma between economic responsibility for both patients and society **[Physician] Concerns on inter-professional relationships** • Risk/fear of conflict or damaging the relationship between various healthcare providers • Unwillingness to change recommendations from secondary/tertiary care • Reluctance to interfere with and/or hesitation to discontinue medications that have been prescribed by a colleague or specialist • GPs may feel a lack of appreciation by secondary/tertiary care colleagues for their role as a GP • Respect for hierarchy **[Physician] Perceptions of pharmacists’ expertise** • Varying perceptions of pharmacists’ recommendations
**Beliefs about Capabilities**	• Self-confidence • Self-esteem • Self-efficacy • Perceived competence • Beliefs	**[Physician] Self-efficacy issues** • Lack of confidence and experience **[Physician] Discrepant beliefs and practice** • Influence from prescriber’s own beliefs, clinical experience and prescribing habits • Respecting prescriber’s right to autonomy **[Patient] Patients’ own expectations and beliefs** • Unrealistic expectations and/or demands from patients and families • Personal beliefs, demands and expectations about their own care and medications • Discrepancies between the patients’ preferences and best practice recommendations • Patients are reluctant or disinclined to stop medications that they have used for a long time • Resistant to change and/or poor acceptance of alternatives • Resistant to non-pharmacological treatment alternatives • Some patients ‘love taking medications’ • Demanding specific medications and when refused, obtaining them from different physicians • Patient’s and family’s wishes for medications • Passive approach adopted by patients
**Optimism**	• Optimism • Pessimism	–
**Beliefs about Consequences**	• Beliefs • Outcome expectancies • Characteristics of outcome expectancies • Anticipated regret • Consequents	**[Physician] Clinical** • Feeling a sense of fear towards older patients in general owing to their frailty and comorbidities • Fear of causing potential harm by deprescribing • Fear of the unknown • Viewing the deprescribing process as a risk to be avoided • Anxiety when the GP’s own conviction conflicts with either that of a specialty of the guidelines • Fear of ‘giving up on the patient’ • Fear of withdrawal effects (e.g. cessation of opioids and benzodiazepines) **[Physician] Social** • Fear of offending other doctors **[Physician] [System] Legal** • Fear of damage to reputation, accountability for adverse outcomes, malpractice or litigation • Litigation fears concerning withholding preventive medications • Fear of medicolegal repercussions or negative responses from patients and their next of kin if rationalizing medications led to clinical events **[Patient] Patients’ own expectations and beliefs** • Unrealistic expectations and/or demands from patients and families • Personal beliefs, demands and expectations about their own care and medications • Discrepancies between the patients’ preferences and best practice recommendations • Resistance to non-pharmacological treatment alternatives • Demanding specific medications and when refused, obtaining them from different physicians • Patient’s and family’s wishes for medications • Passive approach adopted by patients
**Reinforcement**	• Rewards, incentives • Punishment • Reinforcements • Contingencies, sanctions	- Similar to ‘Legal’ concerns in the above ‘Beliefs about Consequences’ domain -
**Intentions**	• Stability of intentions • Stages of change model • Transtheoretical model and stages of change	**[Physician] Inertia and maintaining the status quo** • Differing treatment decisions or changes to the next visit • Easier to maintain the status quo rather than interfere with drug regimes in a stable patient
**Goals**	• Goal / target setting • Goal priority • Action planning	**–**
**Memory, Attention and Decision Processes**	• Memory • Attention • Attention control • Decision making • Cognitive overload / tiredness	**[Physician] Prescribing challenges** • Feeling forced to prescribe • Limited availability of alternatives to medications • Inability to gauge the efficacy effectiveness of a drug for individual patients • Ethical concerns around denying treatments • Need to meet patient expectations • Managing complex drug regimens and side effects • Hesitancy in changing medications that have been prescribed in their current dosage for a long period, or when prescribed by a medical specialist
**Environmental Context and Resources**	• Environmental stressors • Resources / material resources • Organizational culture / climate • Salient events / critical incidents • Person to environment interaction • Barriers and facilitators	**[Physician] [System] Time constraints** • Lack of time to perform medication reviews during the clinic consultation visit • Crowded clinics and high workload, unable to spend too much time with a single patient • Competing demands of practice (e.g. prioritizing other aspects of care rather than deprescribing) • Insufficient time and reimbursement (e.g. to perform medication reviews) **[Physician] [System] Lack of resources** • Lack of access to a pharmacist (e.g. to assist with medication review) • Limited alternative medications • Limited prescribing support (e.g. formularies and computer decision support have limited adaptability and flexibility with multiple conditions) • Lack of resources to assist family caregivers with challenging symptoms (e.g. incontinence) **[System] Lack of inter-professional communication and support** • Lack of communication between prescribers before adding on new drugs • Lack of support from secondary/tertiary care especially with the management of complex patients in general practice **[Physician] [System] Challenges with evidence-based guidelines** • Feeling pressured by guidelines to prescribe medications - including preventive drugs • Less comfortable in deprescribing guideline-recommended therapeutic medications, as compared to deprescribing preventive medications, in patients with poor life expectancy • Easier to pile on the recommendations of one guideline onto another instead of prioritizing • Difficulty in implementing guidelines to older adults with multimorbidity • Exclusion of older adults with multimorbidity in clinical trials • Lack of data for outcomes most important to patients (e.g. improvement in pain control) • Difficulty in applying guidelines because of the heterogeneity of the patients **[System] Fragmentation of care** • Multiple healthcare providers or prescribers • Patients follow up with multiple hospitals and receive medications from multiple providers • Increased specialization in healthcare • Choosing to focus on subspecialty-based care instead of overall management • Fragmentation of care, lack of a specific or unified physician to follow up with • Lack of ownership to assume responsibility for optimizing a specific patient’s care plans **[System] Poor coordination of care** • Lack of coordination/communication between transitions and various levels of care • Lack of access to patients’ clinical data from other healthcare settings • Tough job for coordinating physician • Specialists’ lack of a holistic or geriatric view on older adult patients • Lack of relational continuity of care (e.g. lack of specific/unified physician to follow with) • Attribution of medication management responsibility to other physicians **[System] Information access and documentation** • Lack of coordination of information before adding on new drugs • Lack of or inadequate documentation • Incomplete medication reviews and/or outdated medication lists • Lack of access to information on patients’ current medications • Poor acquisition and documentation of patients’ medication lists • Difficulty in obtaining colleagues’ reasons for prescription • Data lost in the transition from written notes to electronic prescriptions • Lack of access to expert advice and user-friendly decision support (e.g. computer prompts or alerts to notify prescribers of PIMs) **[System] Policy and regulatory issues** • Insufficient reimbursement • Influences of prescribing policy (e.g. perception of managerial meddling and cost cutting) • Quality measure-driven care **[System] Cost issues** • Limited options on insurance formularies **[System] Influences of the pharmaceutical industry** • Widespread marketing of medications in mainstream media • Difficulty in managing direct-to-consumer commercials about drugs and their impact on patients • Physicians themselves may be influenced by pharmaceutical drug representatives
**Social Influences**	• Social pressure and norms • Group conformity / identity • Social comparisons • Group norms • Social support • Power • Intergroup conflict • Alienation • Modelling	**[Patient] Social factors** • Patient’s social context and access to healthcare and resources • Patients who change living or care arrangements may be accompanied by different caregivers to visits, which may result in inconsistent reports from the family and/or lack of continuity of care • Socioeconomic status **[Physician] Health beliefs and culture** • Culture to prescribe more • Prescribing validates illness
**Emotion**	• Fear • Anxiety • Affect • Stress • Depression • Burnout	**[Physician] Anxiety or fear** • Feeling a sense of fear towards older patients in general owing to their frailty and comorbidities • Fear of causing potential harm by deprescribing • Fear of the unknown • Viewing the deprescribing process as a risk to be avoided • Anxiety when the GP’s own conviction conflicts with either that of a specialty or the guidelines • Fear of damage to reputation, accountability for adverse outcomes, malpractice or litigation • Fear of ‘giving up on the patient’ • Fear of offending other doctors • Fear of withdrawal effects (e.g. cessation of opioids and benzodiazepines) • Litigation fears concerning withholding preventative medications • Fear of medico-legal repercussions or negative responses from patients and their next of kin if rationalizing medications led to clinical events **[Physician] Fear of damaging the patient-doctor relationship** • Choosing the maintain the patient-doctor relationship rather than enforce changes or recommendations and threatening that relationship
**Behavioural Regulation**	• Self-monitoring • Breaking habit • Action planning	**–**

^a^*ADE* adverse drug event, *GP* general practitioner, *PIM* potentially inappropriate medications, *PIP* potentially inappropriate prescribing

Our scoping review identified three major stakeholders which influence effective prescribing in older adults – namely the patient, the physician, and the healthcare system at large. By crystallising the barriers into discrete stakeholder profiles, we can shift our perspectives accordingly, highlight specific areas of concern, and help direct further work targeting individual intervention groups. For patients, major themes include poor healthcare literacy, incorrect or misinformed expectations and beliefs, and socioeconomic factors. For physicians, we need to help prescribers navigate the medical complexities in this particular group of patients, equip them with skills on deprescribing in older adults, address concerns regarding interprofessional relationships and role dilemmas, as well as put in place proper safeguards for issues pertaining to negative consequences (e.g. clinical harm and litigation). For the healthcare system, frameworks need to be developed to balance time and resource constraints, improve coordination of care, and establish funding for further research in this area. These findings are summarised in Table 5.

**Table 5:** Barriers to Effective Prescribing in Older Adults – A Summary based on Stakeholders involved

Stakeholder	Domain	Barriers
**Patient**	1) **Knowledge** 2) **Skills** 3) **Beliefs about Capabilities** 4) **Beliefs about Consequences** 5) **Social Influences**	• Lack of knowledge about medications they are taking • Poor healthcare literacy • Non-adherence to medications or visits • Patient’s own expectations and beliefs (e.g. reluctance to discontinue medications, resistance to non-pharmacological treatment) • Social factors (e.g. socioeconomic status, access to healthcare)
**Physician**	1) **Knowledge** 2) **Skills** 3) **Social/Professional Role and Identity** 4) **Beliefs about Capabilities** 5) **Beliefs about Consequences** 6) **Reinforcement** 7) **Intentions** 8) **Memory, Attention and Decision Processes** 9) **Environmental Context and Resources** 10) **Social Influences** 11) **Emotion**	• Medical complexity (e.g. multimorbidity, polypharmacy, increased risk of ADEs) • Lack of knowledge or awareness about PIP • Lack of skills and confidence • Challenges to discussion with patient s (e.g. regarding risk, prognosis and life expectancy) • Paternalistic doctor-patient relationship • Role dilemma (e.g. between economic responsibility for both patients vs. society) • Concerns on inter-professional relationships • Perceptions of pharmacists’ expertise • Self-efficacy issues • Discrepant beliefs and practice • Clinical – fear of causing harm, ‘giving up on the patient’, or withdrawal effects • Social – fear of offending other prescribers • Legal – damage to reputation, accountability issues, medicolegal implications • Inertia and maintaining the status quo • Prescribing challenges (e.g. limited alternatives, managing complex drug regimes • Time constraints • Lack of resources (e.g. limited alternative medications) • Challenges with applicability of evidence-based guidelines in older adults • Health beliefs and culture (e.g. culture to prescribe more) • Anxiety or fear (e.g. fear of the unknown, fear of medicolegal implications) • Fear of damaging the patient-doctor relationship
**Healthcare System**	1) **Environmental Context and Resources**	• Time constraints • Lack of resources (e.g. access to pharmacist, limited prescribing support) • Lack of inter-professional communication and support • Challenges with applicability of evidence-based guidelines in older adults • Fragmentation of care (e.g. increased specialisation, multiple healthcare providers or prescribers) • Poor coordination of care • Information access and documentation (e.g. lack of access to electronic prescriptions) • Policy and regulatory issues (e.g. insufficient reimbursement for medication reviews) • Cost issues (e.g. limited options on insurance formularies) • Influences of the pharmaceutical industry

*ADE* adverse drug event, *PIP* potentially inappropriate prescribing

## Discussion

The TDF elicited multiple domains which both independently and collectively lead to barriers to effective prescribing in older adults in the ambulatory setting, including significant factors pertaining to Knowledge, Skills, Social/Professional Role and Identity, Social Influences and Environmental Context and Resources. We recognise that older adults remain a unique population owing to their medical complexity, multimorbidity and frailty, and this can prove challenging for physicians who lack the knowledge and skillsets to effectively manage this group of patients [48, 49]. Patients and their families may exhibit poor healthcare literacy, ‘doctor-hop’, or express unrealistic expectations including the belief that ‘prescribing validates illness’, and may thus be reluctant to discontinue medications [50]. Contextual factors such as socioeconomic status and access to healthcare and resources must also be considered when examining reasons for non-compliance or discrepant beliefs.

Beyond usual evidence-based guidelines which may be more easily applicable in younger patient groups, there is a constant need to weigh the risks and benefits of each recommendation based on individual patient context in the older adult, and thus no ‘one size fits all’ solution. With increased specialisation and fragmentation of care, physicians have also highlighted concerns regarding inter-professional relationships, hesitancy to interfere with recommendations from secondary or tertiary care, and also fears surrounding adverse outcomes or medicolegal consequences [30, 51]. With limited access to prescribing support or pharmacists in the ambulatory setting, it is thus not surprising that this constant need for debate, consultation and individual patient consideration may be time-consuming, resource-intensive, and thus makes it seemingly easier for physicians to skirt around the issue rather than address PIP, and hope that the decision for effective prescribing may be deferred to the next healthcare provider.

Changing the prescribing climate will thus require interventions targeting multiple stakeholders, including patients, physicians, ambulatory clinic systems and healthcare policy makers. At the level of the community, we need to work towards correcting the misconception that ‘more medications constitute better treatment’, that deprescribing does not equate to ‘giving up on the patient’, and gently reinforce the importance of medication review. Healthcare and social policies need to target the issue of healthcare financing, provision of adequate subsidies and ensuring equal access to healthcare [52]. For physicians, more training and education in managing older adult patients may be helpful, but beyond the equipment of knowledge and skills alone there is also the need to develop good clinical reasoning, which may come with increased exposure to geriatric medicine, delivery of holistic, patient-centred care, and with increased experience and clinical wisdom. It is a delicate process that cannot be rushed and needs to be guided by good role models, alongside provision of adequate support including access to members of the multidisciplinary team (e.g. pharmacists for medication reviews, specialty care nurses for counselling on non-pharmacological management e.g. in the management of urinary incontinence), allowing seamless updating and retrieval of diagnoses and medication lists across institutions and healthcare settings, and encouraging open communication among multiple healthcare providers instead of having each one practise in silo [53–55].

This scoping review distinguishes itself from existing literature in its focus on older adults receiving ambulatory care, which has its own unique set of challenges compared to hospital or residential-based care, as shown in the barriers identified above. Indeed, the original reason for this focus was the anticipation that certain barriers related to environmental context and resources (e.g. time constraints, limited access to a pharmacist, lack of electronic clinical decision support systems) may be more prominent in this setting [56–59]. Moreover, this review constitutes one segment of a wider project that seeks to design and implement a care intervention to improve prescribing for older adults receiving ambulatory care. Thus, it serves as an exploratory piece to better understand the barriers to effective prescribing and maps out these barriers based on the TDF to provide a comprehensive picture on the ambulatory prescribing climate and allow for more systematic development of prospective interventions.

However, because we sought to understand general barriers to prescribing rather than disease-specific or drug-specific considerations, the exclusion of studies that focused on either may have limited the number of studies included in this review. The authors also acknowledge that contextual factors (e.g. access to healthcare) may not be applicable across all healthcare settings, and may need to be interpreted in accordance to each population’s unique needs.

## Conclusion

In conclusion, there exist multiple barriers to effective prescribing which will require multipronged interventions targeting patients, physicians and the healthcare system at large in order to reduce PIP and improve care in older adults. Moving forward, the study team will take findings from this scoping review into a modified Delphi study to explore the significance of the identified TDF domains in Singapore’s context, bearing in mind the potential for cultural and healthcare framework differences between Singapore and the studies included in this review. Building upon empiric evidence for pharmacist involvement in medication reviews, which has demonstrated improvements in prescribing practices and reduction in PIP [60–63], our ultimate aim as a study team would be to develop a physician-pharmacist collaborative care intervention to guide effective prescribing for the older adults in the ambulatory setting.

## Supplementary Information

**Additional file 1.** Search strategy for peer-reviewed data bases

## Data Availability

All data generated or analysed during this study are included in this published article and its supplementary information files.

## Abbreviations

ADE: Adverse drug event
CDSR: Cochrane database of systematic reviews
CINAHL: Cumulative index to nursing and allied health literature
GP: General practitioner
JBI: Joanna Briggs Institute
PIM: Potentially inappropriate medication
PIP: Potentially inappropriate prescribing
PPO: Potential prescribing omission
START: Screening tool to alert doctors to right treatment
STOPP: Screening tool of older person’s prescriptions
TDF: Theoretical domains framework
