# Supplementary material for: Barriers to effective prescribing in older adults: applying the theoretical domains framework in the ambulatory setting – a scoping review
Source: BMC Geriatr. 2020 Nov 9;20:459. doi: 10.1186/s12877-020-01766-7 (PMC7650160; doi:10.1186/s12877-020-01766-7)
Supplement: Supplementary file 1 — Additional file 1. Search strategy for peer-reviewed data bases [file 12877_2020_1766_MOESM1_ESM.docx]

**Additional file 1: Search strategy for peer-reviewed data bases**

| **Databases** | **Search strategy** |
| --- | --- |
| PUBMED | Search (adult[MeSH Terms] OR aged[Text Word] OR seniors[Text Word] OR "older adult"[Text Word] OR "older adults"[Text Word] OR elderly[Text Word] OR older people[Text Word] OR "older person"[Text Word] OR "older persons"[Text Word] OR "older patient"[Text Word] OR "older patients"[Text Word]) AND (inappropriate prescribing[MeSH Terms] OR drug prescriptions[MeSH Terms] OR practice patterns, physician[MeSH Terms] OR prescribing patterns, physician[MeSH Terms] OR polypharmacy[MeSH Terms] OR inappropriate prescribing[Text Word] OR "drug prescription"[Text Word] OR "drug prescriptions"[Text Word] OR "physician practice pattern"[Text Word] OR "physician practice patterns"[Text Word] OR clinical practice pattern[Text Word] OR clinical practice patterns[Text Word] OR prescribing[Text Word] OR deprescribing[Text Word] OR deprescription[MeSH Terms] OR deprescription[Text Word] OR polypharmacy[Text Word]) AND (barrier*[Text Word] OR challenge*[Text Word] OR difficult*[Text Word]) AND (ambulatory care[MeSH Terms] OR primary health care[MeSH Terms] OR outpatient[Text Word] OR "ambulatory care"[Text Word] OR clinic[Text Word] OR clinics[Text Word] OR "primary care"[Text Word] OR "primary healthcare"[Text Word]) |
| CINAHL | (MH adults OR TX ( aged OR elderly OR senior# OR "older adult" OR "older adults" OR "older person" OR "older persons" OR "older people" OR "older patient" OR "older patients" )) AND ((TX ( barrier# or challenge# or difficult* ) AND ( (MH inappropriate prescribing OR MH Prescribing patterns OR MH Practice patterns OR MH polypharmacy OR TX "inappropriate prescribing" OR TX "prescribing patterns" OR TX "practice patterns" OR TX polypharmacy OR TX deprescribing OR TX deprescription# )) AND (MH ( ambulatory care OR outpatient service OR primary health care ) OR TX ( "ambulatory care" OR "outpatient service" OR "primary health care" OR "clinic" )) |
| Embase | (aged or elderly or seniors or "older adult?" or "older patient?" or "older person?" or "older people").af. AND  ((barrier? or challenge? or difficult$) and (inappropriate prescribing or inappropriate prescription? or physician practice pattern? or physician prescribing pattern? or drug prescription? or polypharmacy or deprescribing or deprescription?)).af. AND (ambulatory care or outpatient care or outpatient service? or clinic? or primary care).af. |
| Web of Science | TS= (ambulatory-care or outpatient-care or outpatient-service$ or primary-care or clinic) Indexes=SCI-EXPANDED, SSCI, A&HCI, CPCI-S, CPCI-SSH, ESCI Timespan=All years AND TS= (clinical-practice-pattern$ OR physician-prescribing-pattern$ OR physician-practice-pattern$ OR inappropriate-prescribing OR inappropriate-prescription$ OR drug-prescription$ OR polypharmacy OR deprescribing OR deprescription$) Indexes=SCI-EXPANDED, SSCI, A&HCI, CPCI-S, CPCI-SSH, ESCI Timespan=All years AND TS=(aged or elderly or seniors or older-adult$ or older-patient$ or older-person$ or older-people) Indexes=SCI-EXPANDED, SSCI, A&HCI, CPCI-S, CPCI-SSH, ESCI Timespan=All years |
| Cochcrane | (aged or elderly or seniors or "older adult" OR "older adults" OR "older person" OR "older persons" OR "older people" OR "older patient" OR "older patients") and (barrier? or challenge? or difficult*) and( inappropriate prescribing or drug prescriptions or polypharmacy or practice patterns, physicians)) and (ambulatory care or outpatient care or outpatient service or outpatient services or clinic or clinics) |
